# 足疗程去甲基化药物治疗骨髓增生异常综合征：多中心回顾性研究

**DOI:** 10.3760/cma.j.cn121090-20240405-00124

**Published:** 2024-08

**Authors:** 晓珍 刘, 淑娟 周, 健 黄, 彩芳 赵, 灵旭 蒋, 喻堤 张, 琛 梅, 丽亚 马, 歆平 周, 燕萍 邵, 功强 吴, 希斌 肖, 荣欣 姚, 小红 杜, 通林 胡, 申贤 钱, 园 李, 雪芬 严, 黎 黄, 曼玲 王, 佳萍 傅, 黎红 寿, 文华 江, 伟媚 金, 琳洁 李, 静 乐, 文纪 罗, 蕴 张, 秀杰 周, 浩 张, 香花 郎, 梅 周, 洁 金, 慧芳 蒋, 瑾 张, 桂芳 欧阳, 红艳 佟

**Affiliations:** 1 浙江大学医学院附属第一医院血液科，杭州 310003 Department of Hematology, the First Affiliated Hospital, Zhejiang University School of Medicine, Hangzhou 310003, China; 2 温州医科大学附属第一医院血液科，温州 325015 Department of Hematology, the First Affiliated Hospital of Wenzhou Medical University, Wenzhou 325015, China; 3 浙江大学医学院附属第四医院血液科，义乌 322000 Department of Hematology, the Fourth Affiliated Hospital, Zhejiang University School of Medicine, Yiwu 322000, China; 4 金华市中心医院血液科，金华 321000 Department of Hematology, Jinhua Municipal Central Hospital, Jinhua 321000, China; 5 浙江省台州医院血液科，台州 317000 Department of Hematology, Taizhou Hospital, Taizhou 317000, China; 6 东阳市人民医院血液科，东阳 322100 Department of Hematology, Dongyang Hospital Affiliated to Wenzhou Medical University, Dongyang 322100, China; 7 浙江大学医学院附属第二医院血液科，杭州 310009 Department of Hematology, Zhejiang University School of Medicine Second Affiliated Hospital, Hangzhou 310009, China; 8 温州医科大学附属第二医院血液科，温州 325035 Department of Hematology, The Second Affiliated Hospital of Wenzhou Medical University, Wenzhou 325035, China; 9 宁波市鄞州人民医院血液科，宁波 315040 Department of Hematology, Ningbo Yinzhou People's Hospital, Ningbo 315040, China; 10 浙江省中医院血液科，杭州 310006 Department of Hematology, Zhejiang Provincial Hospital of TCM, Hangzhou 310006, China; 11 杭州市第一人民医院血液科，杭州 310006 Department of Hematology, Hangzhou First People's Hospital, Hangzhou 310006, China; 12 嘉兴市第一医院血液科，嘉兴 314001 Department of Hematology, First Hospital of Jiaxing, Jiaxing 314001, China; 13 衢州市人民医院血液科，衢州 324000 Department of Hematology, Quzhou People's Hospital, Quzhou 324000, China; 14 金华市人民医院血液科，金华 321000 Department of Hematology, Jinhua People's Hospital, Jinhua 321000, China; 15 浙江省人民医院血液科，杭州 310014 Department of Hematology, Zhejiang Provincial People's Hospital, Hangzhou 310014, China; 16 绍兴市人民医院血液科，绍兴 312000 Department of Hematology, Shaoxing People's Hospital, Shaoxing 312000, China; 17 湖州市中心医院血液科，湖州 313000 Department of Hematology, Huzhou Central Hospital, Huzhou 313000, China; 18 台州市第一人民医院血液科，台州 318020 Department of Hematology, Taizhou First People's Hospital, Taizhou 318020, China; 19 丽水市人民医院血液科，丽水 323000 Department of Hematology, Lishui Municipal People Hospital, Lishui 323000, China; 20 丽水市中心医院血液科，丽水 323000 Department of Hematology, Lishui Central Hospital and Fifth Affiliated Hospital of Wenzhou Medical University, Lishui 323000, China; 21 宁波市医疗中心李惠利医院血液科，宁波 315040 Department of Hematology, Li Huili Hospital, Ningbo 315040, China; 22 杭州市萧山区第一人民医院血液科，杭州 311200 Department of Hematology, The First People's Hospital of Xiaoshan District, Hangzhou 311200, China; 23 乐清市人民医院血液科，乐清 325600 Department of Hematology, Yueqing People's Hospital, Wenzhou 325600, China; 24 海宁市人民医院血液科，海宁 314400 Department of Hematology, Haining People's Hospital, Haining 314400, China; 25 瑞安市人民医院血液科，瑞安 325200 Department of Hematology, The Third Affiliated Hospital of Wenzhou Medical University, Rui'an 325200, China; 26 永康市第一人民医院血液科，永康 321300 Department of Hematology, Yongkang First People's Hospital, Yongkang 321300, China; 27 诸暨市人民医院血液科，诸暨 311800 Department of Hematology, Zhuji People's Hospital, Zhuji 311800, China; 28 浙江省立同德医院血液科，杭州 310012 Department of Hematology, Tongde Hospital of Zhejiang Province, Hangzhou 310012, China; 29 浙江大学医学院附属邵逸夫医院血液科，杭州 310016 Department of Hematology, Run Run Shaw Hospital Affiliated to Zhejiang University School of Medicine, Hangzhou 310016, China; 30 宁波大学附属第一医院血液科，宁波 315010 Department of Hematology, The First Affiliated Hospital of Ningbo University School of Medicine, Ningbo 315010, China

**Keywords:** 骨髓增生异常综合征, 去甲基化药物, 地西他滨, 阿扎胞苷, 预后, Myelodysplastic syndromes, Hypomethylating agents, Decitabine, Azacitidine, Prognosis

## Abstract

**目的:**

研究去甲基化药物（HMA）足疗程治疗骨髓增生异常综合征（MDS）的疗效和安全性。

**方法:**

纳入来自浙江省45家医院的409例接受了至少连续4个周期的HMA单药起始治疗的MDS患者，评估HMA疗效和安全性。采用Mann-Whitney *U*或卡方检验比较组间临床资料差异，采用Logistic回归和Cox回归分析疗效与生存的影响因素，采用Kaplan-Meier法进行生存分析。

**结果:**

409例患者HMA治疗的中位疗程为6（4～25）个疗程。完全缓解（CR）率为33.98％，总缓解率（ORR）为77.02％。多因素分析显示，复杂核型（*P*＝0.02，*OR*＝0.39，95％*CI* 0.18～0.84）是CR的独立良好影响因素；TP53突变（*P*＝0.02，*OR*＝0.22，95％*CI* 0.06～0.77）是ORR的独立良好预测因素。患者的中位总生存（OS）时间为25.67（95％*CI* 21.14～30.19）个月，HMA治疗有反应（*P*＝0.036，*HR*＝0.47，95％*CI* 0.23～0.95）是OS的独立良好预后因素，而伴有复杂核型（*P*＝0.024，*HR*＝2.14，95％*CI* 1.10～4.15）、发生白血病转化（*P*<0.001，*HR*＝2.84，95％*CI* 1.64～4.92）、TP53突变（*P*＝0.012，*HR*＝2.19，95％*CI* 1.19～4.07）均是OS的独立不良预后因素。HMA减剂量较标准剂量在疗效和中位OS时间均无显著差异。地西他滨和阿扎胞苷治疗组的CR率、ORR均无显著差异。接受地西他滨治疗患者的中位OS时间长于接受阿扎胞苷治疗患者（29.53个月对20.17个月，*P*＝0.007），但地西他滨组严重骨髓抑制、肺炎发生率高于阿扎胞苷组。

**结论:**

在患者能够耐受情况下，连续规律地使用适当剂量的HMA，有利于MDS患者最大程度从治疗中获益。

## Abstract (en)

**Objective:**

To evaluate the efficacy and safety of hypomethylating agents（HMA）in patients with myelodysplastic syndromes（MDS）.

**Methods:**

A total of 409 MDS patients from 45 hospitals in Zhejiang province who received at least four consecutive cycles of HMA monotherapy as initial therapy were enrolled to evaluate the efficacy and safety of HMA. Mann-Whitney *U* or Chi-square tests were used to compare the differences in the clinical data. Logistic regression and Cox regression were used to analyze the factors affecting efficacy and survival. Kaplan-Meier was used for survival analysis.

**Results:**

Patients received HMA treatment for a median of 6 cycles（range, 4–25 cycles）. The complete remission（CR）rate was 33.98％ and the overall response rate（ORR）was 77.02％. Multivariate analysis revealed that complex karyotype（*P*＝0.02, *OR*＝0.39, 95％*CI* 0.18–0.84）was an independent favorable factor for CR rate. TP53 mutation（*P*＝0.02, *OR*＝0.22, 95％*CI* 0.06–0.77）was a predictive factor for a higher ORR. The median OS for the HMA-treated patients was 25.67（95％*CI* 21.14–30.19）months. HMA response（*P*＝0.036, *HR*＝0.47, 95％*CI* 0.23–0.95）was an independent favorable prognostic factor, whereas complex karyotype（*P*＝0.024, *HR*＝2.14, 95％*CI* 1.10–4.15）, leukemia transformation（*P*<0.001, *HR*＝2.839, 95％*CI* 1.64–4.92）, and TP53 mutation（*P*＝0.012, *HR*＝2.19, 95％*CI* 1.19–4.07）were independent adverse prognostic factors. There was no significant difference in efficacy and survival between the reduced and standard doses of HMA. The CR rate and ORR of MDS patients treated with decitabine and azacitidine were not significantly different. The median OS of patients treated with decitabine was longer compared with that of patients treated with azacitidine（29.53 months *vs* 20.17 months, *P*＝0.007）. The incidence of bone marrow suppression and pneumonia in the decitabine group was higher compared with that in the azacitidine group.

**Conclusion:**

Continuous and regular use of appropriate doses of hypomethylating agents may benefit MDS patients to the greatest extent if it is tolerated.

骨髓增生异常综合征（MDS）是一种起源于造血干/祖细胞的克隆性恶性疾病，好发于老年人，其特征是骨髓无效造血导致血细胞减少，高风险向急性髓系白血病（AML）转化（转白）[1]–[4]。近年来，二代测序（NGS）技术在MDS的基础研究和临床实践中都取得了突破性进展[5]–[6]。

去甲基化药物（HMA）是较高危MDS患者一线治疗药物[4],[7]。美国食品药品监督管理局（FDA）于2004年批准阿扎胞苷（AZA）用于治疗MDS，一项随机Ⅲ期研究表明，与常规治疗相比，AZA可延长较高危MDS患者的总生存（OS）期[8]。地西他滨（DEC）是AZA的脱氧核苷酸类似物，在2006年被FDA批准用于MDS的治疗[9]。多项临床试验显示，DEC或AZA治疗MDS，完全缓解（CR）率为7％～35％，中位OS期为16～24个月[8]–[14]。然而，一些真实世界研究的报道显示，HMA单药治疗的CR率仅为10％～15％，中位OS期为10～16个月[15]–[22]，远低于临床试验的疗效。由于HMA治疗需要4～6个疗程才能获得最佳治疗反应[7],[23]–[25]，在真实世界中一些患者往往使用的疗程较少，影响了疗效。那么，足疗程的患者中是否能从HMA的治疗中获益？本研究通过回顾性分析浙江省45家医院409例接受至少4个疗程HMA治疗的MDS患者的临床数据，旨在探讨接受足疗程HMA治疗的MDS患者的疗效和生存获益情况。

## 病例与方法

一、病例资料

本研究在浙江省45家医院开展，回顾性纳入2010年3月至2021年6月确诊为MDS的409例患者。纳入标准：①确诊为MDS且接受至少4个疗程HMA单药治疗；②HMA使用标准剂量或减剂量方案，每个周期DEC连续治疗5 d，AZA连续治疗7 d；③基线资料和随访资料完整，诊断时必须具有骨髓常规、骨髓活检、免疫分型和染色体报告，可评估最佳疗效和生存状态。排除标准：①合并其他活动性恶性肿瘤；②既往接受过细胞毒治疗；③入组过临床试验；④HMA治疗的前4个疗程中，疗程间隔时间超过2个月。

根据维也纳最低诊断标准[26]–[27]及2016年版WHO造血及淋巴组织肿瘤诊断标准[28]对MDS进行诊断及分型：MDS伴单系发育异常（MDS-SLD）13例（3.18％），MDS伴多系发育异常（MDS-MLD）59例（14.43％），MDS伴环形铁粒幼红细胞（MDS-RS）10例（2.44％），MDS伴原始细胞增多-1（MDS-EB-1）182例（44.50％），MDS伴原始细胞增多-2（MDS-EB-2）139例（33.99％），MDS伴单纯del（5q）1例（0.24％），MDS未分类（MDS-U）5例（1.22％）。采用修订版国际预后评分系统（IPSS-R）[29]对患者进行预后分层：极低危组2例（0.49％）、低危组33例（8.07％）、中危组110例（26.89％）、高危组157例（38.39％）、极高危组107例（26.16％）。

二、治疗方案

①AZA标准剂量：75 mg·m^−2^·d^−1^，第1～7天，皮下注射，28 d为1个疗程，直至疾病进展。②AZA减低剂量：100 mg/d，第1～7天，皮下注射，28 d为1个疗程，直至疾病进展。③DEC标准剂量：20 mg·m^−2^·d^−1^，静脉滴注，第1～5天，28 d为1个疗程，直至疾病进展。④DEC减低剂量：25 mg/d，静脉滴注，第1～5天，28 d为1个疗程，直至疾病进展。

患者发生疾病进展或转白后，根据主管医师判断进行治疗方案调整，包括：①白血病样化疗，高三尖杉酯碱、阿克拉霉素、去甲氧柔红霉素、阿糖胞苷等药物组合方案；②HMA联合bcl-2抑制剂维奈克拉；③异基因造血干细胞移植（allo-HSCT）；④最佳支持治疗等。

三、染色体核型分析

骨髓细胞经过24 h培养，收集细胞常规制片，对至少20个骨髓细胞的中期分裂象进行R显带，根据《人类细胞遗传学国际命名体制（ISCN2013）》[30]描述核型异常。按照IPSS-R染色体核型分组标准[29]进行染色体核型预后分组。

四、NGS基因检测

共有171例患者在HMA治疗起始之前进行了NGS检测。由于不同医院NGS检测涵盖的血液肿瘤相关基因数量不同，对有共同检测数据的20个常见基因突变情况进行了采集并分析，包括TP53、TET2、ASXL1、RUNX1、SF3B1、SRSF2、U2AF1、DNMT3A、BCOR、NPM1、WT1、EZH2、CBL、IDH1、ZRSR2、ETV6、NF1、JAK2、STAG2、IDH2。测序原始数据利用人类基因组数据库（HG19）、COSMIC、PolyPhen-2等数据库进行生物信息学分析，筛选致病性基因突变位点。

五、疗效及不良反应评估

根据2006国际工作组（IWG2006）标准评估治疗最佳反应[31]。总有效率（ORR）定义为CR率、部分缓解（PR）率、骨髓完全缓解伴血液学改善（mCR+HI）率、骨髓完全缓解（mCR）率、血液学改善（HI）率的总和。将疾病稳定（SD）和治疗失败定义为治疗无反应。不良事件评估采用不良事件通用术语标准（CTCAE5.0）[32]。

六、随访

所有患者都采用门诊或电话联系的方式进行随访，末次随访时间为2023年2月。OS期定义为从HMA治疗起始至患者死亡、失访或末次随访日期。对于接受allo-HSCT的患者，OS期定义为HMA治疗起始至移植日期。

七、统计学处理

采用SPSS 26.0、GraphPad Prism 8和R 4.1.2进行统计学分析。计量资料以中位数（范围）表示，组间比较采用Mann-Whitney *U*检验。计数资料以例数（构成比）表示，组间比较采用卡方检验或Fisher确切概率法。通过Logistic回归分析获得影响HMA治疗反应的独立因素。通过建立OS的Cox回归模型获得影响HMA预后的独立因素。采用Kaplan-Meier法进行生存分析。双侧*P*<0.05为差异有统计学意义。

## 结果

一、患者临床特征

本研究共纳入409例患者（表1）。诊断时的中位年龄为66（22～91）岁，男女比为1.62∶1（253∶156）。初诊时中位ANC为1（0～18）×10^9^/L，中位HGB为73（30～170）g/L，中位PLT为53.5（2～618）×10^9^/L，中位骨髓原始细胞比例为7.0（0～19.5）％，65例（15.89％）患者携带复杂核型。78.49％的患者为MDS伴原始细胞增多型，其中MDS-EB-1型占44.50％，MDS-EB-2型占33.99％。根据IPSS-R评分，83.40％的患者在初诊时为较高危MDS。

**表1:** 409例骨髓增生异常综合征（MDS）患者的基线临床特征

指标	总队列 （409例）	地西他滨组 （253例）	阿扎胞苷组 （156例）	*P*值
年龄[岁，*M*（范围）]	66（22~91）	64（22~91）	67.5（31~89）	0.02
性别（例，男/女）	253/156	164/89	89/67	0.11
WBC[×10^9^/L，*M*（范围）]	2.3（0.5~29.5）	2.5（0.5~29.5）	2.3（0.6~23.2）	0.06
ANC[×10^9^/L，*M*（范围）]	1.0（0~18.0）	1.1（0.2~18.0）	0.9（0~13.8）	0.09
HGB[g/L，*M*（范围）]	73（30~170）	73（30~170）	72（30~170）	0.45
PLT[×10^9^/L，*M*（范围）]	53.5（2~618）	54（2~566）	49（3~618）	0.44
血清铁蛋白[µg/L，*M*（范围）]	450（5~4 445）	450（8~4 445）	427（5~3678）	0.92
骨髓原始细胞比例[%，*M*（范围）]	7.0（0~19.5）	7.0（0~19.0）	7.0（0~19.5）	0.87
复杂核型[例（%）]	65（15.89）	46（18.18）	19（12.18）	0.11
中位疗程[个，*M*（范围）]	6（4~25）	5（4~24）	6（4~25）	0.65
WHO2016分型[例（%）]				0.07
MDS-SLD	13（3.18）	9（3.56）	4（2.56）	
MDS-MLD	59（14.43）	33（13.04）	26（16.67）	
MDS-RS-SLD	7（1.71）	5（1.98）	2（1.28）	
MDS-RS-MLD	3（0.73）	1（0.40）	2（1.28）	
MDS伴单纯del（5q）	1（0.24）	0	1（0.64）	
MDS-EB-1	182（44.50）	113（44.66）	69（44.23）	
MDS-EB-2	139（33.99）	89（35.18）	50（32.06）	
MDS-未分类	5（1.22）	3（1.18）	2（1.28）	
IPSS-R风险分层[例（%）]				0.73
极低危	2（0.49）	1（0.40）	1（0.64）	
低危	33（8.07）	20（7.90）	13（8.33）	
中危	110（26.89）	71（28.06）	39（25.00）	
高危	157（38.39）	91（35.97）	66（42.31）	
极高危	107（26.16）	70（27.67）	37（23.72）	

**注** MDS-SLD：MDS伴单系血细胞发育异常；MDS-MLD：MDS伴多系血细胞发育异常；MDS-RS-SLD：MDS伴环状铁粒幼红细胞单系血细胞发育异常；MDS-RS-MLD：MDS伴环状铁粒幼红细胞多系血细胞发育异常；MDS-EB-1：MDS伴原始细胞增多-1型；MDS-EB-2：MDS伴原始细胞增多-2型；IPSS-R：骨髓增生异常综合征修订国际预后积分系统

所有患者接受HMA中位疗程数为6（4～25）个疗程，其中接受DEC治疗的患者253例，接受AZA治疗的患者156例。253例接受DEC治疗的患者中，使用标准剂量共86例，使用减低剂量共167例。156例接受AZA治疗患者中，使用标准剂量共41例，使用减低剂量共115例。HMA标准剂量组和减低剂量组基线临床特征如表2所示，标准剂量组患者中位年龄为62岁，而减低剂量组患者中位年龄为67岁（*P*＝0.01）。治疗中187例（45.72％）患者出现疾病进展，其中120例（64.17％）接受了白血病样化疗，57例（30.48％）接受了HMA联合维奈克拉治疗，10例（5.35％）仅接受了支持治疗。409例患者中，33例（8.07％）接受了allo-HSCT。

**表2:** 接受标准剂量和减低剂量去甲基化药物（HMA）治疗患者的基线临床特征

指标	HMA标准剂量组 （127例）	HMA减低剂量组 （282例）	*P*值
年龄[岁，*M*（范围）]	62（22~85）	67（23~91）	0.010
性别（例，男/女）	89/38	164/118	0.020
WBC [×10^9^/L，*M*（范围）]	2.4（0.6~19.5）	2.3（0.5~29.5）	0.533
ANC [×10^9^/L，*M*（范围）]	1.0（0~13.8）	1.0（0~18.0）	0.636
HGB [g/L，*M*（范围）]	74.5（30~170）	72（34~159）	0.384
PLT [×10^9^/L，*M*（范围）]	47（2~564）	55（3~618）	0.169
血清铁蛋白[µg/L，*M*（范围）]	439（5~3 381）	450（26~4 445）	0.526
骨髓原始细胞[%，*M*（范围）]	8.0（0~18.0）	7.0（0~19.5）	0.063
复杂核型[例（%）]	20（15.75）	45（15.96）	0.957
中位疗程[个，*M*（范围）]	5（4~21）	6（4~25）	0.786
WHO2016分型[例（%）]			0.252
MDS-SLD	3（2.36）	10（3.55）	
MDS-MLD	15（11.81）	44（15.60）	
MDS-RS-SLD	0	7（2.48）	
MDS-RS-MLD	0	3（1.06）	
MDS伴单纯del（5q）	0	1（0.35）	
MDS-EB-1	58（45.67）	124（43.97）	
MDS-EB-2	48（37.80）	91（32.27）	
MDS-未分类	3（2.36）	2（0.72）	
IPSS-R风险分层[例（%）]			0.028
极低危	1（0.79）	1（0.35）	
低危	5（3.94）	28（9.93）	
中危	44（34.65）	66（23.40）	
高危	41（32.28）	116（41.13）	
极高危	36（28.34）	71（25.18）	

**注** MDS-SLD：MDS伴单系血细胞发育异常；MDS-MLD：MDS伴多系血细胞发育异常；MDS-RS-SLD：MDS伴环状铁粒幼红细胞单系血细胞发育异常；MDS-RS-MLD：MDS伴环状铁粒幼红细胞多系血细胞发育异常；MDS-EB-1：MDS伴原始细胞增多-1型；MDS-EB-2：MDS伴原始细胞增多-2型；IPSS-R：骨髓增生异常综合征修订国际预后积分系统

409例患者中171例在诊断时完成了基因突变NGS检测。其中，158例（92.4％）至少有1个基因存在体细胞突变。最常见的突变基因为TP53（25％）、ASXL1（24％）、TET2（22％）、SRSF2（13％）、RUNX1（11％）和SF3B1（11％）。

二、治疗反应

409例患者HMA治疗的ORR为77.02％（315/409），其中CR 139例（33.98％），PR 7例（1.39％），mCR+HI 63例（15.41％），mCR 54例（13.21％），HI 52例（12.71％），SD 64例（15.65％），治疗失败30例（7.33％）。

对比DEC或AZA治疗的CR率（37.15％对28.85％，*P*＝0.09）、DEC标准剂量组和减低剂量组CR率（34.88％对38.32％，*P*＝0.60）、AZA标准剂量组和减低剂量组CR率（19.51％对32.17％，*P*＝0.12），差异均无统计学意义。将年龄、性别、血细胞计数、血清铁蛋白、骨髓原始细胞比例、是否伴有复杂核型、HMA药物、WHO诊断分型、IPSS-R预后分组、基因突变等因素对CR率的影响进行单因素分析，结果显示，年龄（*P*＝0.09）、复杂核型（*P*＝0.01）、HMA药物（*P*＝0.09）、TP53突变（*P*＝0.05）对CR率有影响。将上述4个*P*<0.1的因素纳入多因素分析，仅复杂核型（*P*＝0.02，*OR*＝0.39，95％*CI* 0.18～0.84）与较高的CR率相关，携带复杂核型的患者CR率达到47.69％（31/65），而TP53突变（*P*＝0.63，*OR*＝0.80，95％*CI* 0.32～1.99）对CR率无独立影响意义。

对比DEC和AZA治疗组的ORR（79.45％对73.08％，*P*＝0.14）、DEC标准剂量组和减剂量组的ORR（81.40％对78.44％，*P*＝0.58）、AZA标准剂量组和减剂量组的ORR（68.30％对74.78％，*P*＝0.42），差异均无统计学意义。对年龄、性别、血细胞计数、血清铁蛋白、骨髓原始细胞比例、是否复杂核型、HMA药物、WHO诊断分型、IPSS-R预后分组、基因突变等因素对ORR的影响进行单因素分析，结果显示，骨髓原始细胞比例（*P*＝0.08）、TP53突变（*P*＝0.02）对ORR有影响。将上述2个*P*<0.1的因素纳入多因素分析显示，仅TP53突变（*P*＝0.02，*OR*＝0.22，95％*CI* 0.06～0.77）是ORR的独立良好预测因素，携带TP53突变的患者ORR高达92.68％（38/41）。

三、生存分析

截至末次随访，中位随访20个月，共有161例（39.36％）患者死亡，总体中位OS期为25.67（95％*CI* 21.14～30.19）个月。单因素分析结果显示，HGB（*P*＝0.001）、PLT（*P*＝0.004）、骨髓原始细胞比例（*P*＝0.04）、IPSS-R预后分组（*P*＝0.02）、是否复杂核型（*P*<0.001）、HMA药物（*P*＝0.01）、HMA治疗是否有反应（*P*＝0.005）、是否白血病转化（*P*<0.001）、是否STAG2突变（*P*＝0.09）、是否SRSF2突变（*P*＝0.06）和是否TP53突变（*P*＝0.04）影响患者OS。将上述单因素分析*P*<0.1的11个因素纳入多因素Cox回归模型中，结果显示，HMA治疗有反应（*P*＝0.036，*HR*＝0.47，95％*CI* 0.23～0.95）是独立良好预后因素，而伴有复杂核型（*P*＝0.024，*HR*＝2.14，95％*CI* 1.10～4.15）、发生白血病转化（*P*<0.001，*HR*＝2.839，95％*CI* 1.64～4.92）、TP53突变（*P*＝0.012，*HR*＝2.19，95％*CI* 1.19～4.07）均是独立不良预后因素。伴有复杂核型的患者中位OS期为13.83（95％*CI* 12.03～15.63）个月，较不伴复杂核型患者的28.23（95％*CI* 24.32～32.14）个月显著缩短（*P*<0.001）（图1A）。HMA治疗无反应患者的中位OS期为18.07（95％*CI* 13.83～22.31）个月，较HMA治疗有反应患者的27.90（95％*CI* 23.14～32.66）个月显著缩短（*P*＝0.005）（图1B）。发生AML转化的患者中位OS为20.70（95％*CI* 18.65～22.75）个月，显著短于未转白患者的39.60（95％*CI* 27.95～51.25）个月（*P*<0.001）（图1C）。TP53突变患者的中位OS期显著短于TP53野生型患者［22.00（95％*CI* 7.48～36.52）个月对28.23（95％*CI* 21.56～34.90）个月，*P*＝0.04］（图1D）。

**图1:**
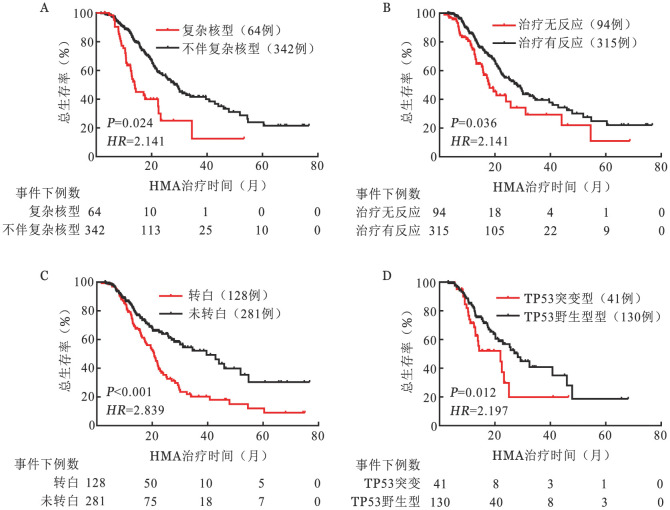
接受足疗程去甲基化药物（HMA）治疗骨髓增生异常综合征（MDS）患者复杂核型（A）、治疗反应（B）、白血病转化（转白）（C）、TP53突变（D）对总生存的影响

亚组分析显示，接受DEC治疗患者的OS期长于接受AZA治疗患者［29.53（95％*CI* 24.26～34.80）个月对20.17（95％*CI* 15.92～24.42）个月，*P*＝0.007］（图2A）。然而，接受DEC标准剂量和减低剂量的患者中位OS期差异无统计学意义［29.23（95％*CI* 22.87～35.59）个月对29.77（95％*CI* 18.92～40.62）个月，*P*＝0.655］（图2B）。同样，接受AZA标准剂量和减低剂量的患者中位OS期差异也无统计学意义［21.23（95％*CI* 4.07～76.80）个月对19.77（95％ *CI* 11.78～30.68）个月，*P*＝0.524］（图2C）。

**图2:**
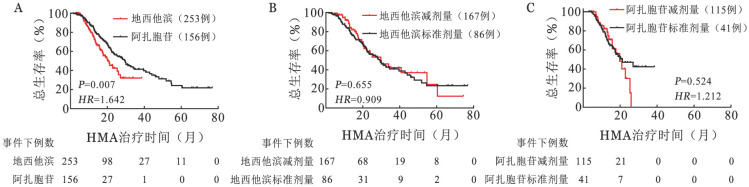
去甲基化药物（HMA）不同治疗药物和不同剂量治疗骨髓增生异常综合征（MDS）患者的生存比较 **A** 地西他滨与阿扎胞苷组；**B** 地西他滨标准剂量与减剂量；**C** 阿扎胞苷标准剂量与减剂量

四、不良反应

所有患者中，3～4级血液学不良反应包括：白细胞减少345例（84.40％）、中性粒细胞减少363例（88.8％）、贫血356例（87％）、血小板减少334例（81.70％）。DEC组3～4级血液学不良反应比例略高于AZA组，白细胞减少比例差异有统计学意义（90.20％对81.50％，*P*＝0.01），而中性粒细胞减少（93.90％对89.90％，*P*＝0.15）、贫血（91.50％对85.50％，*P*＝0.06）、血小板减少（90.60％对89.80％，*P*＝0.81）比例差异均无统计学意义。

3～4级非血液学不良反应中，最常见的是肺炎，发生率为15.16％（62/409），DEC组3～4级肺炎发生率显著高于AZA组（20.60％对6.40％，*P*<0.001）。其他常见非血液学不良反应还有乏力19例（4.60％）、软组织感染16例（3.90％）、颅内出血5例（1.20％）。在1～2级非血液学不良反应中，最常见的是乏力（58.70％），比较常见的有肺炎（42.10％）、恶心呕吐（39.60％）、肝功能损害（17.80％）。

## 讨论

由于MDS异质性极大，不同患者病程与预后差异明显[1],[33]–[34]。HMA包括DEC和AZA，主要通过表观调控发挥去甲基化作用，激活抑癌基因[7],[24],[35]，是较高危MDS患者的一线治疗药物。多项研究结果显示，HMA治疗MDS的CR率为7％～35％，ORR为40％～60％，中位OS期为16～24月[8]–[12],[36]。在针对中国MDS患者的研究中，一项多中心Ⅱ期研究结果显示，较高危MDS患者接受AZA标准剂量治疗，53％患者达到HI，中位OS期为22个月[37]。另一项多中心Ⅲb期临床研究结果显示，DEC治疗MDS的CR率为9.8％，ORR为63.3％，中位OS期为20.6个月[38]。然而，在一些真实世界研究中，HMA单药治疗的CR率仅为10％～15％，中位OS期为10～16个月[15]–[17],[21]。由于评估HMA最佳治疗反应需要4～6个疗程，治疗的不充分会影响HMA疗效，本队列纳入了至少连续4个疗程接受HMA单药治疗的MDS患者，以研究足疗程HMA治疗的获益。409例MDS患者的中位疗程数为6个，总体CR率为33.98％，ORR为77.02％，中位OS期为25.97个月，与既往研究[8]–[10],[15]–[17],[20]–[22],[36]–[41]相比显示出较好的治疗反应。既往一项多中心观察性研究也显示，足疗程DEC治疗MDS患者的ORR为62.9％，CR率为27.3％，1年OS率80.9％，2年OS率为60.9％[42]。提示足够的疗程很重要。

关于DEC和AZA疗效差异，多项研究报道两者总体疗效相当。一项来自韩国的回顾性研究显示，DEC和AZA的ORR分别为63.5％和52％（*P*＝0.155），2年OS率分别为42.2％和42.1％（*P*＝0.944）[43]。另一项来自韩国的观察性研究表明，AZA和DEC的ORR（44％对52％）、中位OS期（26个月对22.9个月）和1年时白血病转化率（16％对22％）差异均有统计学意义[44]。在一项MDS-RAEB的回顾性研究中，AZA和DEC治疗的ORR（49.1％对64.5％，*P*＝0.166）、中位OS期（20.4个月对16.8个月，*P*＝0.59）差异均无统计学意义[45]。另一项MDS-RAEB的回顾性研究也证实了上述结果[22]。最近一项回顾性研究中，629例MDS患者根据IWG 2023标准[46]评估疗效，AZA和DEC单药治疗的CR率分别为14.8％和5.6％，ORR分别为48.6％和50.4％，差异无统计学意义[47]。本研究中DEC和AZA治疗MDS的CR率、ORR差异均无统计学意义。但是本研究数据显示，接受DEC治疗的患者比接受AZA治疗的患者有更长的OS期，中位OS期分别为29.53个月和20.17个月（*P*＝0.007），推测这可能与DEC组患者更年轻有关，DEC组患者中位年龄为64岁，而AZA组患者中位年龄为67.5岁（*P*＝0.02）。

本研究显示，标准剂量和减低剂量的HMA在治疗反应率及生存时间上差异均无统计学意义。既往也有研究报道，相较于标准剂量，减低剂量HMA治疗患者更能生存获益[11],[48]–[49]。由于患者在接受标准剂量的持续治疗中可能会造成严重骨髓抑制及其他相关不良反应，影响患者耐受性造成治疗的中断或增加死亡风险，因此，针对骨髓抑制严重及耐受性差的患者适当地进行减量治疗，可以保证患者获得足够的疗程及持续的给药，让患者获得最佳的治疗反应及最大程度地从治疗中获益。

本研究多因素分析发现，复杂核型与较高的CR率相关，TP53突变是ORR的独立良好预测因素。虽然携带复杂核型及TP53突变的患者有早期良好的治疗反应，但从生存影响因素看，复杂核型、白血病转化、TP53突变均是独立不良预后因素，仅HMA治疗有反应是独立良好预后因素。既往研究同样表明，携带TP53突变的患者虽然对HMA有较好的反应[25],[50]，但患者生存预后很差[35],[51]–[53]，因此建议携带TP53突变尤其合并复杂核型的患者应积极入组临床试验或进行HSCT。

本研究显示，HMA治疗的主要不良反应为3～4级血细胞减少和肺炎，其他不良反应如乏力、软组织感染、颅内出血等发生率较低，DEC的严重粒细胞减少和肺炎发生率高于AZA，与文献报道类似[8],[15],[44]。

总之，通过本队列的回顾性研究表明，接受至少4个周期HMA治疗的MDS患者有较好的疗效和预后，且HMA的剂量调整不影响疗效及生存。对于治疗不耐受的患者，可适当调整剂量，让患者尽量获得HMA持续治疗，以获得最大的疗效和生存获益。此外，本研究显示，DEC与AZA在CR率和ORR上差异无统计学意义，接受DEC治疗的患者生存时间虽然更长，但其不良反应尤其是肺炎发生率更高。由于MDS是一种异质性克隆性疾病，在临床实践中需要根据患者的年龄、疾病状态、合并症等综合判断选择合适的HMA治疗。
